# Clinical manifestations in female carriers of mucopolysaccharidosis type II: a spanish cross-sectional study

**DOI:** 10.1186/1750-1172-8-92

**Published:** 2013-06-25

**Authors:** Encarna Guillén-Navarro, María Rosario Domingo-Jiménez, Carlos Alcalde-Martín, Ramón Cancho-Candela, María Luz Couce, Enrique Galán-Gómez, Olga Alonso-Luengo

**Affiliations:** 1 Unidad de Genética Médica. Servicio de Pediatría, Hospital Clínico Universitario Virgen de la Arrixaca, El Palmar, Murcia, Spain; 2 Sección de Neuropediatría, Hospital Clínico Universitario Virgen de la Arrixaca, El Palmar, Murcia, Spain; 3 Servicio de Pediatría, Hospital Universitario Rio Hortega, Valladolid, Spain; 4 Unidad de Errores Innatos del Metabolismo. Servicio de Neonatología, Hospital Clínico Universitario de Santiago de Compostela, Santiago de Compostela, Spain; 5 Unidad de Genética Clínica. Servicio de Pediatría. Hospital Materno Infantil Infanta Cristina. Facultad de Medicina, Universidad de Extremadura, Badajoz. Centro de Investigación Biomédica en Red de Enfermedades Raras (CIBERER. U724), Badajoz, Spain; 6 Sección de Neuropediatría, Hospital Universitario Virgen del Rocio, Sevilla, Spain

**Keywords:** Hunter syndrome, Mucopolysaccharidosis type II, Carriers, Heterozygotes, X- inactivation, Iduronate 2-sulfatase, Glycosaminoglycans

## Abstract

**Background:**

Mucopolysaccharidosis type II (MPS II) is an inherited X-linked disease associated with a deficiency in the enzyme iduronate 2-sulfatase due to iduronate 2-sulfatase gene (*IDS*) mutations. Recent studies in MPS II carriers did not find clinical involvement, but these were mainly performed by anamnesis and patients’ self-reported description of signs and symptoms. So although it is rare in heterozygous carriers, investigations in other types of inherited X-linked disorders suggest that some clinical manifestations may be a possibility. The aim of this study was to evaluate the clinical pattern in female carriers of MPS II and to determine whether clinical symptoms were associated with the X-chromosome inactivation (XCI) pattern and age.

**Methods:**

Female carriers of MPS II were genetically identified by molecular analysis of *IDS*. The clinical evaluation protocol included pedigree analysis, a comprehensive anamnesis, complete physical examination, ophthalmological evaluation, brain-evoked auditory response, electrocardiogram, echocardiogram, pulmonary function tests, abdominal sonogram, skeletal survey, neurophysiological studies, blood cell counts and biochemistry, urine glycosaminoglycan (GAGs) quantification, karyotype and pattern of XCI.

**Results:**

Ten women were included in the study. The mean age of the participants was 40.2 ± 13.1 years. Six carriers presented a skewed XCI pattern, 3 of whom (aged 38, 42 and 52 years) had increased levels of GAGs in the urine and showed typical MPS II clinical manifestations, such as skeletal anomalies, liver abnormalities, carpal tunnel syndrome, recurrent ear infection, hypoacusia and more frequent severe odontological problems without coarse facial features.

**Conclusions:**

This is the first study performing a comprehensive evaluation of heterozygous MPS II carriers. Our results provide evidence of possible progressive, age-dependent, mild clinical manifestations in MPS II female carriers with a skewed XCI pattern, most likely affecting the normal allele. Further comparative studies with systematized clinical examinations in larger age-stratified populations of MPS II female carriers are required.

## Background

Mucopolysaccharidosis type II (MPS II; OMIM 309900), or Hunter syndrome, is one of the most common types of X-linked recessive inherited disorder with an estimated incidence between 1:110,000 and 135,500 live births [1,2]. It is caused by a deficiency in the activity of the enzyme iduronate 2-sulfatase (I2S; EC 3.1.6.13) that results from a mutation in the iduronate 2-sulfatase gene (*IDS*). The subsequent lack of glycosaminoglycan (GAGs) catabolism leads to an accumulation of dermatan sulfate and heparan sulfate in the lysosomes of many tissues, which makes MPS II a multisystem disease. MPS II includes the following main manifestations: short stature, dysostosis multiplex, joint contractures, coarse facies, hepatosplenomegaly, deafness, recurrent ear and sinopulmonary infections, obstructive airway disease, valve abnormalities and in several cases, mental impairment. Therefore, its management requires a multidisciplinary approach and specific enzyme replacement therapy, which is now available and which may change the natural history of the disease [3].

MPS II is an X-linked recessive condition, in which female patients are infrequent. Moreover, in MPS II the X chromosome carrying the mutant allele is expected to be preferentially inactivated in some cells like chondrocytes or hepatocytes. Consequently, MPS II heterozygous subjects are rarely affected [4]. X-chromosome inactivation (XCI) was investigated first by Lyon, who postulated that only one of the X chromosomes was active in females, with the inactivation of either the maternal or paternal X-chromosome alleles occurring in a random and irreversible process [5].

Although it could be expected that females present 50:50 of active maternally and paternally derived X chromosomes after XCI, the most common ratios are 60:40 or 70:30 [6]. Moderately skewed XCI, defined as 75:25, or severely skewed inactivation (90:10 or over), could be pathogenic and could be associated with advanced age [7].

The presence of two alleles carrying pathological mutations or a structural X-chromosome abnormality simultaneously leads to symptomatic female heterozygotes [8]. To date, 12 case reports of females affected with MPS II have been described in the literature; in 10 of these cases, the disease was associated with skewed XCI [9-18].

Recently, several clinical studies were designed to identify any clinical manifestations in heterozygous female carriers of MPS II. No evidence of clinical involvement was found, despite lower plasma and leukocyte I2S activities [8,19]. Nevertheless, these investigations were limited due to a lack of more sensitive clinical investigations. When a systematic analysis was undertaken in other inherited X linked diseases, such as Fabry disease or Barth syndrome, clinical signs and abnormal biochemical results were frequently found in carriers [20,21].

In contrast to previous studies in which the collection of the data was mainly performed by anamnesis and patients’ self-reported description of sign and symptoms, the aim of this cross-sectional study was to evaluate those clinical features using a broad panel of clinical examinations and complementary tests in ten female carriers of MPS II. In addition, we evaluated clinical symptoms according to the XCI pattern and age of participants.

## Methods

We included heterozygous mothers and other heterozygous female relatives of MPS II male patients, previously identified by genetic analysis, included in the Spanish Hunter Outcome Survey (HOS) who were being seen at participating centers. HOS is a worldwide multi-center observational long-term follow-up registry, sponsored by Shire HGT, which is open to all patients diagnosed with the disease [22]. This registry was established in 2005 to assess the natural history of Hunter syndrome and the safety and effectiveness of enzyme replacement therapy with idursulfase. Our study was approved by the local Institutional Ethics Committee of the Hospital Clínico Universitario Virgen de la Arrixaca in Murcia, Spain. Informed consent was obtained from all participants before beginning the study evaluation.

The clinical data were collected during carriers’ visits to the centers by five clinicians from the Hunter Spanish Expert Council, a group of reference in the diagnosis and management of MPS II in Spain. Comprehensive anamneses were obtained, including information regarding the age or degree of relationship with the proband. A systematical investigation on the extent and distribution of physical involvement in female carriers of MPS II was conducted in 10 women using standardized clinical examination protocols. Complete physical examination by systems and organs was performed including full ophthalmological evaluation, brain-evoked auditory response, electrocardiogram, echocardiogram, pulmonary function tests, abdominal sonogram, skeletal survey, cognitive assessment and neurophysiological studies (such as median nerve electrophysiological study). A symptomatic carrier should have the characteristic signs and symptoms classically associated to MPS II. The following additional analyses were also performed: hematimetry, general biochemistry (including liver enzymes), quantification of GAGs in urine, karyotypes and XCI studies.

Total GAGs were measured according to an improved dimethylmethylene blue (DMB) test [23]. The normal range in adults for this method was 1.6 ± 0.8 mg GAGs/mmol creatinine.

The cytogenetic analysis was performed on metaphase chromosomes obtained from phytohemagglutinin (PHA)-stimulated peripheral blood lymphocytes following standard procedures. All chromosome preparations were G-banded and analyzed with a minimum resolution of 550 to 600 bands [24].

The X-inactivation pattern was determined from peripheral lymphocytes by polymerase chain reaction (PCR) analysis of a polymorphic CAG trinucleotide repeat in the polyglutamine region of the androgen receptor (AR) gene [25]. The XCI pattern was defined as skewed with a cut-off value of ≥75:25 of the X-inactivation ratio (moderately skewed inactivation) and ≥90:10 (severely skewed), and the 50:50 cut-off value was defined as random [7,26].

## Results

Ten female carriers of MPS II from 6 unrelated families with no parental consanguinity participated in the study. The mean age of the participants was 40.5 ± 13.1 and ranged from 17 to 67 years. The majority of the participating women were mothers (n = 6) of previously diagnosed MPS II patients recruited in the HOS registry, but there was also one grandmother, one aunt, one sister and one cousin.

The carriers’ information at baseline, including medical history and/or physical examination, is presented in Additional file 1: Table S1 and is split into two groups according to the patients’ age (≥40 or <40). Globally, missense point mutations in *IDS* were seen in five families and a small deletion in one. Only two of the missense mutations were previously described (c.998C>T and c.1048A>C). The karyotypes were normal in all women (data not shown). Extreme skewing XCI pattern was not identified in any of them. Moderately skewed XCI pattern was found in 6 women, of whom 4 were 40 years or older. Three out of six had increased GAGs. Therefore, a conjunction of skewed XCI pattern and GAGs elevation was observed in 2 / 5 aged carriers (≥ 40 years) and 1 / 5 in the group (< 40 years).

The results of the clinical examinations and complementary examinations are also described in Additional file 1: Table S1. None of them showed coarse facial features. Six female carriers presented overweight or obesity. Three carriers, aged 38, 42 and 52, showed moderately skewed XCI pattern (75:25, 81:19 and 86:14 respectively), increased GAGs levels and clinically significant Hunter disease manifestations, especially in the older ones. For example, female number 1 (52 years old) presented dentures since her twenties, recurrent acute otitis, eardrum perforation, bilateral neurosensorial deafness, bronchopathy, hepatomegaly, neurological and skeletal findings, and mild to moderate carpal tunnel syndrome (CTS). Skeletal anomalies, short stature and CTS were predominant symptoms in female carrier number 2 (42 year). Female number 6 (38 years) showed important odontological problems, recurrent otitis media, bilateral neurosensorial hypoacusia and mild skeletal manifestations with precocious degenerative spine changes.

Overall, the laboratory data revealed that platelets were increased up to 4.83 × 10^5^ in 5 / 9 carriers (Data not shown). The neurophysiological examination detected 3 / 5 women with mild to moderate CTS. The cognitive assessment identified 5 / 7 females with normal intelligence quotient (IQ) scores, one with a low IQ and the oldest female carrier with Hunter manifestations who presented a limited borderline score, according to the Kaufman Brief Intelligence test and the Wechsler Intelligence Scale for Adults. These two females were from the same family.

**Figure 1:**
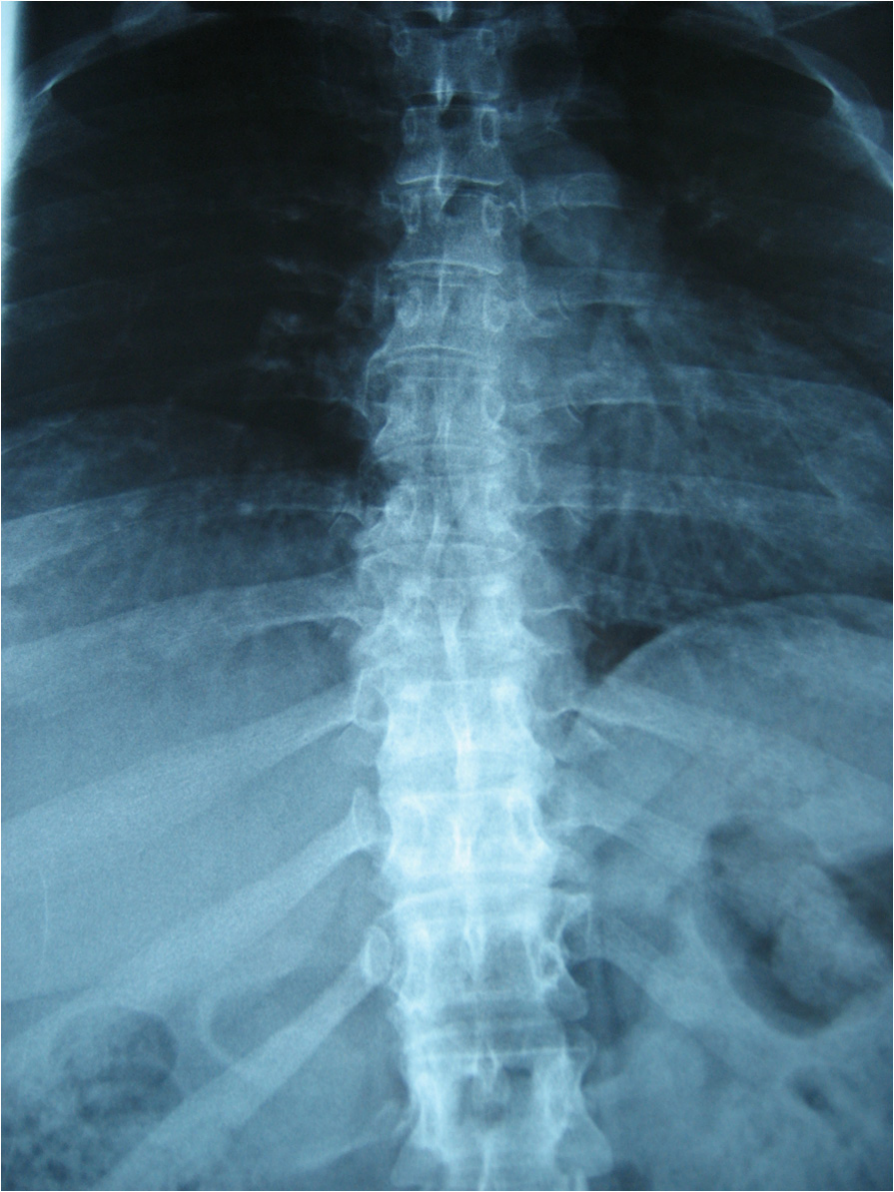
Paddle-shaped ribs with narrowing at the vertebral end.

Echographics and imaging exams were not performed in all women for technical reasons. Abdominal imaging was obtained from 6 participants, with hepatic anomalies observed in 3 who also showed other Hunter manifestations (1 mild hepatomegaly, 2 mild hepatic steatosis). All 3 carriers with increased GAG levels presented degenerative bone changes. The 42-year old carrier (number 2) showed hip dysplasia and typical Hunter paddle-shaped ribs (Figure 1 X-ray image). Except for a minimal aortic insufficiency with dilatation in the oldest participant with increased levels of GAGs (number 1), none of the rest of the participants presented abnormalities in echocardiogram or electrocardiogram. Brain MRI was performed in eight carriers and was normal in all of them.

## Discussion

As many X-linked diseases, in MPS II has been assumed that female carriers remain asymptomatic. Moreover, recent publications have found no evidence for clinical involvement in heterozygous female carriers, although there was a slightly lower *IDS* activity in plasma and leukocytes compared to non-heterozygotes [8,19]. Nevertheless, in carriers of other recessive inherited X-linked disorders, such as Fabry disease [20] or Barth syndrome [21], systematic explorations showed some clinical manifestations, which were not previously described. In contrast to previous studies, our results show that the presence of clinical manifestations might be not uncommon in MPS II female heterozygous when a comprehensive evaluation is performed. Moreover, some of these women may exhibit typical clinical features of the disease. The most frequent symptomatology found in this study has been precocious odontological problems, bone anomalies, respiratory and ear problems, CTS and hepatic anomalies.

In MPS II, over 300 types of mutations in the *IDS* gene have been identified [27], most of them are private and missense mutations. In our study, a missense mutation was observed in 5 / 6 families and a novel mutation in 4 / 6 families. There may be a correlation between genotype / phenotype, so that severity of the mutation could have an impact in the way the disease is clinically manifested. For example, the mutation in carrier 6 is severe as it leads to a protein frameshift and may be associated with an earlier symptomatology.

Together with the I2S enzyme activity and *IDS* mutation test, GAGs quantification in urine is the gold standard for the diagnosis of MPS II. We used the method described by Stone et al., which is widely used in the literature [23]. Three of 10 participants showed elevated GAGs levels in urine.

First described by Lyon [5], XCI or lyonization appears to be involved in many X-linked transmitting processes in female carriers, such as MPS II. The variability in X inactivation produces milder clinical phenotypes in females, often more variable than in males [5,28,29]. Although for some authors skewed XCI was essential in the phenotype expression in heterozygotes of X-linked diseases and particularly in Fabry disease [30-32], others did not find a correlation with phenotype or severity, concluding that XCI in leukocytes of females with Fabry disease was not useful for predicting prognosis and should not be used to define therapeutic options [33]. In a normal situation, a random X inactivation of the same allele in 50% of cells (50:50) or 60:40 is expected [6], but a skewing pattern, defined as a inactivation ≥ 75:25, may lead to X-linked pathologies even in women [20]. In our results, 6 / 10 female carriers of MPS II showed skewed XCI pattern. This pattern was also described in previously published clinical case reports in females [9-18] and represents a higher rate than expected for healthy female population. As per Salsano et al. investigation on X-linked adrenoleukodystrophy, moderately skewed XCI was found in 22.4% of healthy subjects in the control group [26] and Sharp et al. obtained a rate of 16% in normal women over 60 years old [34]. Some other investigations additionally described that symptomatic heterozygotes usually present skewed X inactivation with a predominant expression of the mutated allele [35]. This was a limitation in our study, as we did not determine the allele that was preferentially inactivated, similarly to the de Camargo investigation [19]. Although no significant correlation has been found between the levels of leukocyte *IDS* activity and the levels of urinary GAGs in MPS II carriers [8], the occurrence of clinical manifestations in some of our carriers presented both skewed inactivation and elevated GAGs suggesting that the mutated allele might be preferentially expressed [8]. The most frequent and significant clinical anomalies were bone changes and mild to moderate CTS and neurosensorial hypoacusia, identified by specific complementary examinations (skeletal survey, median nerve electrophysiological study and brain-evoked auditory response), not by patient-reported anamnesis.

Subjects were divided according to their age, in order to evaluate whether symptomatology was stronger at older ages, because it is known that the proportion of females showing a highly skewed pattern of X inactivation increases markedly with age. Life expectancy has been recently established in around 80 years old (84,57 years old) [36], therefore we select the age of 40 years old to divide subjects into groups and ensure we gather both first and last vital periods of women.

XCI increases in tissues with age, reaching up to 40% of skewed lyonization in women aged 60 or over [6]. The skewing process does not seem to reflect a single heritable genetic locus, but rather corresponds to a complex trait determined by the combinatorial effect of stochastic events and selection biases occurring after primary XCI, caused by X-linked allelic variants of genes affecting cellular growth [37]. The age-skewing process has been described in several investigations, but the clinical significance of this finding is still uncertain [26,37-39]. According to our results, the mean age of the patients was 40.5 ± 13.1 years, which was slightly older compared with other investigations (Schwartz et al.: 35.5 ± 8.6 years [8]; de Camargo et al.: 36.1 ± 7.6 years [19]). Four of the 5 women aged 40 or over showed moderately skewed inactivation, 2 of which had a greater symptomatology in conjunction with elevated GAG levels. In heterozygotes for X-linked disorders, the degree of skewing is expected to be correlated with the degree to which females present symptoms of the X-linked disease [8]. As explained before, in some X-linked disorders, such as Fabry disease, a consistent relationship between phenotype and XCI in blood cells could not be confirmed and it was hypothesized that other cell tissues should be more appropriate for the analysis [33]. In our results, the severity of the symptoms is greater in the oldest carrier with higher skewing XCI pattern female carrier 1 with 52 years (86:14), followed by female 2 with 42 years (81:19) and female 3 with 38 years (75:25). Interestingly, female carrier 1 had a normal abdominal ultrasound 4 years before which also might suggest the progression of the symptoms with age.

Unfortunately, I2S enzyme activity was not measured in these women. It is already known that MPS II carriers show lower plasma and leukocyte I2S activities when compared with no carriers but this reduction was not associated, in Schwartz study, either with increased levels of urinary GAGs or with the occurrence of clinical manifestations [8]. Furthermore, they pointed out that I2S activity measured in other cell types, such as chondrocytes or hepatocytes, should be more relevant to evaluate the progression of the disease.

Pinto et al. explained that the major differences in clinical manifestations among X-linked diseases may depend on the cell autonomy of the gene product involved and, therefore, on the occurrence of cross-correction mechanism, which was deficient in Fabry disease and preserved in MPS II [4]. It was suggested that MPS symptomatic heterozygotes would present skewed X-inactivation with predominant expression of the mutant allele [4].

Previous investigations on MPS II were based on data from medical history and patients’ anamnesis about signs and symptoms in organs and systems typically involved in MPS II [8,19]. In the work of de Camargo et al., the anamnesis was focused on cardiologic, pulmonary, bone, and gastric problems, while the complementary exams included brain magnetic resonance imaging, liver, spleen, and spine computed tomography scans [19]. Additionally, they determined plasma and leukocyte *IDS* activity, urinary levels of GAGs, karyotyping and conducted an X-inactivation assay [19]. Also, Schwartz et al. collected the medical history and performed the physical examination according to expected MPS II symptomatology; and included a leukocyte IDS activity and GAGs determination in 24-hour urine samples [8]. Therefore, little data is available on cognitive and neurophysiological alterations, which may not be sufficient to identify a subtle clinical symptomatology. This is the first study performing a comprehensive evaluation of heterozygous MPS II carriers, in where a complete physical examination was conducted individually by system and organ, including a full ophthalmological evaluation, brain-evoked auditory response, electrocardiogram, echocardiogram, pulmonary function tests, abdominal sonogram, skeletal survey, cognitive assessment and neurophysiological studies (such as median nerve electrophysiological study).

We found certain clinical signs in the whole group of carriers, such as CTS diagnosed in 4 / 6 women and mild hepatic pathologies described in 3 / 6 participants. Additionally, moderate to severe periodontal disease or dental abscess in 6 / 9 participants and an elevation of platelets was observed in 5 / 9 participants. We are not aware of previous reports of platelet alterations associated with this type of mucopolysaccharidosis and certainly we cannot describe the practical significance of this finding.

Overall, we found 3 carriers aged 38, 42 and 52 showing some typical MPS II clinical manifestations, including recurrent acute otitis, bilateral neurosensorial deafness, dentature since their twenties, hepatomegaly, skeletal anomalies and mild to moderate CTS, skewed XCI and GAGs elevation. Subjects aged 42 and 52 years (number 1 and 2, respectively) are sisters and signs and symptoms of the disease were more clearly evident in the older women, so it is possible that MPS II’s penetrance and clinical severity index in female carriers increase with age.

It would have been interesting to study also non-heterozygotes females as part of a control group, because some of these clinical findings may be commonly found in healthy population. Nevertheless, the work was conducted inside the public health care system and we were limited given the costly and comprehensive work-up to be done in females without added risk factors (such as MPS II carriers). The small population of our study makes it difficult to reach a conclusive result, but our data provide traces of subtle clinical and biochemical signs in female carriers of MPS II.

## Conclusions

These results suggest that, like other X-linked diseases, MPS II heterozygotes may show a progressive disease phenotype, and this appears to be associated with a significant skewing of X-inactivation, presumably in the normal allele. The evolution of signs and symptoms is often a better indicator of MPS II involvement than a static snapshot of the presence or absence of certain manifestations. Therefore, it is important to monitor changes over time. It would be convenient that MPS heterozygotes are monitored closely at least every 12–24 months thereafter at a centre with experience. Assessments should include evaluation of the musculoskeletal and cardiovascular systems, ears, airways, eyes, skin, nervous system, abdomen and gastrointestinal system, as outlined in Additional file 1: Table S1. More frequent assessment might be necessary in those in whom signs and symptoms are progressing and eventually the available treatment options should be discussed in symptomatic heterozygous females.

However, further comprehensive studies that look for subtle signs and symptoms of this disease are required in a larger age-stratified female carrier population.

## Abbreviations

AR: Androgen receptor; BAEPs: Brainstem auditory evoked potentials; CTS: Carpal tunnel syndrome; DMB: Dimethylene blue; GAG: Glycosaminoglycans; HOS: Hunter Outcome Survey; IDS: Iduronate 2-sulfatase gene; IQ: Intelligence quotient; I2S: Enzyme iduronate 2-sulfatase; MPS II: Mucopolysaccharidosis type II; MRI: Magnetic resonance imaging; PCR: Polymerase chain reaction; XCI: X chromosome inactivation.

## Competing interests

E G-N, MR D-J, C A-M, R C-C, ML C, E G-G, and O A-L are members of the Spanish HOS (Hunter Outcome Survey), which receives financial support from Shire Human Genetic Therapies. E G-N is a member of the HSEEC (Hunter Syndrome European Expert Council), which receives financial support from Shire Human Genetic Therapies. Except for the editorial assistance acknowledged below, the authors did not receive any financial support for the project presented in this manuscript.

## Authors’ contributions

EG-N has contributed in the study design, recruitment of participants and in the collection of their data, as well as in the analysis and interpretation of results. EG-N also participated in drafting the paper and has revised the intellectual content to give final approval of the manuscript. MRD-J, CA-M, RC-C, MLC, EG-G, and OA-L were involved in the recruitment of subjects, data collection and analysis of results. All authors have critically read and revised the paper, and approved the final version to be published.

## Supplementary Material

Additional file 1: Table S1 Clinical, biochemical and genetic data in MPS II female carriers. Click here for file
