# Supplementary material for: Clinical manifestations in female carriers of mucopolysaccharidosis type II: a spanish cross-sectional study
Source: Orphanet J Rare Dis. 2013 Jun 25;8:92. doi: 10.1186/1750-1172-8-92 (PMC3697996; doi:10.1186/1750-1172-8-92)
Supplement: Additional file 1: Table S1 — Clinical, biochemical and genetic data in MPS II female carriers. [file 1750-1172-8-92-S1.docx]

|  |  | **Female carrier number** | | | | | | | | | | |
| --- | --- | --- | --- | --- | --- | --- | --- | --- | --- | --- | --- | --- |
|  |  | **1** | **2** | | **3** | **4** | **5** | **6** | **7** | **8** | **9** | **10** |
| **Age, years** |  | Age ≥ 40 | | | | | | Age < 40 | | | | |
|  |  | 52 | | 42 | 43 | 40 | 67 | 38 | 39 | 34 | 30 | 17 |
| **Genetic and biochemical evaluation** | **IDS gene mutation** | c.925A>G  p.T309A | | c.925A>G  p.T309A | c.998C>T  p.G374sp | c.454G>C  p.R110S | c.1048A>C  p.N350H | c.457del  p.W153GfsX60 | c.135G>A  p.D45E | c.1048A>C  p.N350H | c.925A>G  p.T309A | c.925A>G  p.T309A |
|  | **XCI pattern** | Skewed | | Skewed | Skewed | Skewed | Random | Skewed | Skewed | Random | Random | Random |
|  | **Inactivation percentage** | 86:14 | | 81:19 | 75:25 | 80:20 | 59:41 | 75:25 | 85:15 | 54:46 | 53:47 | 52:48 |
|  | **GAG determination** | 3.54*(↑) | | 3.41*(↑) | 1.71* | 2.0* | 1.93* | 6.24*(↑) | 1.43* | 1,60* | 1.54* | 1.94* |
| **Somatometry** | **Height, cm (p)** | 156.5 (p10) | | 147 (p<1) | 168 (p75) | 160 (p26) | NA | 166 (p63) | 164 (p50) | 164 (p50) | 159 (p21) | 151 (p2) |
|  | **Weight, kg (p)** | 62 (p69) | | 73.4 (p96) | 61 (p65) | 62 (p69) | 73 (p96) | 127.7 (p>99) | 58 (p51) | 90 (p>99) | 108.8 (p>99) | 54.7 (p34) |
|  | **Head circumference, cm (p)** | 55.5 (p50) | | 55 (p36) | 55 (p36) | 53 (p4) | NA | 57 (p86) | 54 (p14) | 57.8 (p95) | 57 (p86) | 52.5 (p2) |
| **Ophthalmological** | **Bilateral cortical opacity** | + | | - | - | - | - | - | - | - | - | - |
|  | **Sclerosis of crystalline** | + | | - | - | - | - | - | - | - | - | - |
|  | **Myopia magna** | - | | - | - | - | - | + | - | - | - | + |
|  | **Retinal degeneration** | - | | - | - | - | - | + | - | - | - | - |
|  | **Fibrotic neovascular membrane** | - | | - | - | - | - | + | - | - | - | - |
|  | **Retinal detachment** | - | | - | - | - | - | + | - | - | - | - |
|  | **Conjuntivae tumor** | + | | - | - | - | - | - | - | - | - | - |
|  | **Eye surgery** | + | | - | - | - | - | + | - | - | - | - |
|  | **Crystalline luxation** | - | | - | - | - | - | + | - | - | - | - |
| **Odontological** | **Delayed/abnormal dentition** | - | | - | - | - | - | + | - | + | - | + |
|  | **Dental abscess** | - | | - | - | - | + | + | - | + | - | - |
|  | **Periodontal disease** | + | | - | - | - | - | - | - | - | + | - |
|  | **Dentures at early age** | + | | - | - | - | - | + | - | - | - | - |
|  | **Gingival hypertrophy** | - | | - | - | - | - | + | - | - | - | - |
| **ENT (Ear-Nose-Throat)** | **Nose obstruction** | - | | - | - | - | - | - | - | - | + | - |
|  | **Otitis media** | + | | - | - | - | - | + | - | + | + | - |
|  | **Eardrum perforation** | + | | - | - | - | - | - | - | - | - | - |
|  | **Ear drainage** | - | | - | - | - | - | - | - | + | - | - |
|  | **BAEPs- Neurosensorial bilateral hypoacusia** | + | | - | - | - | - | + | - | - | - | - |
|  | **Bilateral myringoplasty** | + | | - | - | - | - | - | - | - | - | - |
|  | **Adeno-amygdalectomy** | - | | - | - | - | - | - | - | + | - | - |
| **Respiratory** | **Bronchopathy** | + | | - | - | - | + | - | - | - | - | - |
|  | **Apnea-dysnea** | - | | - | - | - | + | + | - | - | - | - |
|  | **Asthma** | - | | - | - | - | - | - | + | - | - | - |
|  | **Pulmonary Function Test** | NA | | NA | N | N | NA | N | A** | NA | N | NA |
| **Gastroenterological** | **Difficulty swallowing** | - | | - | - | - | - | + | - | - | - | - |
|  | **Gastritis** | - | | - | - | - | - | - | - | - | + | - |
|  | **Gastric surgery (by-pass)** | + | | - | - | - | - | - | - | - | - | - |
|  | **Constipation** | + | | - | - | - | - | - | - | + | - | - |
| **Bone anomalies** | **Bone pain (Neck, back, articular and/or hand pain)** | - | | + | - | - | + | - | - | + | + | - |
|  | **Kyphosis** | - | | - | - |  | - | + | - | - | - | - |
|  | **Hip dysplasia** | - | | + | - | - | - | - | - | - | - | - |
|  | **Pelvic and femoral osteotomies** | - | | + | - | - | - | - | - | - | - | - |
|  | **Prosthesis in the knee** | - | | + | - | - | - | - | - | - | - | - |
|  | **Hallux valgus (bunion)** | + | | - | - | - | - | - | - | - |  | - |
|  | **Limited hand mobility** | + | | - | - | - | - | - | - | - | - | - |
| **Skeletal survey** | **Scoliosis** | + | | + | - | - | NA | - | - | - | - | + |
|  | **Spine degenerative changes** | - | | - | - | - | NA | + | - | - | - | - |
|  | **Pectus carinatum** | + | | - | - | - | NA | - | - | - | - | - |
|  | **Mild paddle-shaped ribs** | - | | + | - | - | NA | - | - | - | - | - |
|  | **Right hip ankylosis and femoral luxation** | - | | + | - | - | NA | - | - | - | - | - |
|  | **Irregularity at distal area of radius and degenerative changes at radiocarpal and intercarpal joints** | + | | - | - | - | NA | - | - | - | - | - |
| **Neurological** | **Muscle weakness** | - | | - | - | - | - | + | - | - | - | - |
|  | **Anxiety and depression** | - | | - | - | - | + | + | - | + | - | - |
|  | **Neurosis** | + | | - | - | - | - | - | - | - | - | - |
|  | **Vertigo** | - | | - | - | - | + | + | - | - | - | - |
|  | **Numbness in hands** | - | | - | - | - | - | - | - | + | + | - |
|  | **Carpal tunnel syndrome***** | + | | + | - | - | NA | - | - | NA | + | - |
| **Metabolic** | **Diabetes** | + | | - | - | - | - | - | - | - | - | - |
|  | **Hyperthyroidism** | - | | - | - | + | - | - | - | - | - | - |
|  | **Obesity** | + | | - | - | - | - | + | - | + | + | - |
|  | **Hypertension** | - | | - | - | - | + | - | - | - | - | - |
| **Others findings** | **Urinary tract infection (UTI)** | - | | + | - | - | - | - | - | - | + | - |
|  | **Bening breast tumor** | - | | - | + | - | + | - | - |  | - | - |
|  | **Lymphoma** | - | | - | - | - | + | - | - | - | - | - |
| **Abdominal Sonogram** | **Mild hepatomegaly** | + | | - | NA | - | NA | - | NA | NA | - | - |
|  | **Hepatic steatosis** | - | | - | NA | - | NA | + | NA | NA | + | - |
| **Echocardiogram** | **Aortic insufficiency with dilatation** | + | | - | - | - | NA | - | - | NA | - | - |
| **Cerebral MRI** |  | N | | N | N | N | NA | N | N | NA | N | N |
| **Cognitive assessment** | **Kaufman Brief Intelligence Test (K-BIT).** | N-low (IQ: 88) | | N (IQ: 95) | - | N (IQ>90) | NA | N | - | NA | NA | - |
|  | **Wechsler Adult Intelligence Scale (WAIS).** | - | | - | N (IQ: 103) | - | NA | - | N (IQ: 99) | NA | NA | Limited (IQ: 73) |
|  |  |  | |  |  |  |  |  |  |  |  |  |
| *Normal values are 1.6 ± 0.8 mg GAG/mmol creatinine | | | |  |  |  |  |  |  |  |  |  |
| ** Mild Obstructive Pattern | |  | |  |  |  |  |  |  |  |  |  |
| *** Median nerve electrophysiological study | |  | |  |  |  |  |  |  |  |  |  |
| (↑): elevated; "-": Absence; "+": Presence | |  | |  |  |  |  |  |  |  |  |  |
| N: Normal values, A: Abnormal values, NA: Not available | | | |  |  |  |  |  |  |  |  |  |
| BAEPs: Brainstem auditory evoked potentials, GAG: Glycosaminoglycans, IDS: Iduronate 2-sulfatase gene, MRI: Magnetic resonance imaging, P: percentile, XCI: X chromosome inactivation | | | | | | | | | | | | |
